# miR-34a is involved in CSE-induced apoptosis of human pulmonary microvascular endothelial cells by targeting Notch-1 receptor protein

**DOI:** 10.1186/s12931-018-0722-2

**Published:** 2018-01-26

**Authors:** Ying-Jiao Long, Xiao-Peng Liu, Shan-Shan Chen, Dan-Dan Zong, Yan Chen, Ping Chen

**Affiliations:** 1 Division of Respiratory Medicine, The Second Xiangya Hospital, Central South University, Changsha, Hunan 410011 China; 2 Research Unit of Respiratory Disease, Central South University, Changsha, Hunan 410011 China; 3 Diagnosis and Treatment Center of Respiratory Disease, Central South University, Changsha, Hunan 410011 China; 4 Department of Intensive Care Unit, The Want Want Hospital, Changsha, Hunan 410013 China; 5 Department of Radiology, The Second Xiangya Hospital, Central South University, Changsha, Hunan 410011 China; 6 Division of Respiratory Medicine, Department of Internal Medicine, The Second Xiangya Hospital, Central South University, No.139 Middle Renmin Road, Changsha, Hunan 410011 China

**Keywords:** miR-34a, Cigarette smoke extract, Apoptosis, Vascular endothelial cells, Notch-1

## Abstract

**Background:**

Abnormal apoptosis of lung endothelial cells has been observed in emphysematous lung tissue and has been suggested to be an important upstream event in the pathogenesis of chronic obstructive pulmonary disease (COPD). Studies have shown that microRNAs (miRNAs) contribute to the pathogenesis of pulmonary diseases by regulating cell apoptosis. The present study was designed to investigate the expression of microRNA-34a (miR-34a) in human pulmonary microvascular endothelial cells (HPMECs) exposed to cigarette smoke extract (CSE), and the potential regulatory role of miR-34a in endothelial cell apoptosis.

**Results:**

Our results showed that the expression of miR-34a was significantly increased in CSE-treated HPMECs, and inhibiting miR-34a attenuated CSE-induced HPMEC apoptosis. Furthermore, expression of Notch-1, a receptor protein in the Notch signalling pathway, was decreased and was inversely correlated with miR-34a expression in HPMECs treated with CSE. Computational miRNA target prediction confirmed that Notch-1 is a target of miR-34a. Luciferase reporter assay further confirmed the direct interaction between miR-34a and the 3’-untranslated region (UTR) of Notch-1. Restoration of Notch-1 pathway was able to partially block the effect of miR-34a on HPMEC apoptosis. These results indicate that Notch-1 is a critical downstream target of miR-34a in regulating the CSE-induced HPMEC apoptosis.

**Conclusions:**

Our results suggest that miR-34a plays a key role in CSE-induced endothelial cell apoptosis by directly regulating its target gene Notch-1 in endothelial cells.

## Background

Chronic obstructive pulmonary disease (COPD) is a common cause of disability and mortality worldwide. Over 300 million people suffer from COPD. It is currently the fourth leading cause of death worldwide and predicted by the World Health Organization to become the third leading cause by 2030 [1]. The apoptosis of structural cells in the lung has recently been suggested to be an important upstream event in the pathogenesis of COPD [2–7]. Our previous studies demonstrated that apoptotic cells, including alveolar epithelial cells and pulmonary endothelial cells, were present in greater numbers in COPD lungs than normal lungs. Moreover, cigarette smoke extract (CSE) induced apoptosis dose-dependently and time-dependently in human umbilical vein endothelial cells [8–10]. Cigarette smoke is well known to be a predominant risk factor for pulmonary diseases including COPD and contains free radicals and oxidative compounds, which are highly mutagenic [11].

MicroRNAs (miRNAs) are a conserved class of post-transcriptional regulators that modulate gene expression by binding to complementary sequences in the coding or the 3′-untranslated region (UTR) of target mRNAs [12]. Several studies have recently shown that exposure to cigarette smoke in both humans and rats lead to global alterations in miRNA expression [11, 13]. Shiro Mizuno et al. described miRNAs as modulators of smoking-induced gene expression changes in patients with COPD, and reported that microRNA-34a (miR-34a) and miR-199a-5p levels were significantly increased in COPD lung tissues and strongly associated with FEV1% predicted [14]. In TSA (trichostatin A)-treated emphysematous rat lungs and human pulmonary microvascular endothelial cells (HPMEC), miR-34a expression was significantly increased [15]. Current research has revealed that ectopic over-expression of miR-34a can induce cell cycle arrest, apoptosis, and senescence in malignant cells by directly targeting mRNA [16, 17]. Moreover, dysregulation of miR-34a expression inhibited angiogenesis as well as endothelial cell functions [18]. However, the mechanism by which miR-34a regulates apoptosis of pulmonary endothelial cells in COPD remains unclear.

In the present study, we investigated the role of miR-34a in CSE-induced apoptosis of HPMECs. We assessed the expression level of miR-34a in cell lines and examined its effects on HPMEC apoptosis by detecting the rate of apoptosis and the expression of apoptotic proteins. Furthermore, we explored the target genes miR-34a and the underlying mechanism of its function. This study will provide a better understanding of the pathogenesis of COPD.

## Methods

### Cell lines and cell culture

HPMECs, the primary microvascular endothelial cells derived from the lungs of the human foetus, were obtained from Sciencell Research Laboratories (San Diego, CA; 3000). HPMECs were grown in 95% air and 5% CO_2_ at 37 °C in specific endothelial cell medium according to the manufacturer’s protocol. Cells were passaged at 80% confluence and grown to full confluence for the experiments. All experiments were performed in triplicate and repeated at least three times. At the end of the incubation period, cell lysates and culture supernatants were harvested and stored at − 80 °C until further analysis.

After serum starvation for 24 h, HPMEC medium was supplemented with CSE at the indicated concentrations.

### Preparation of CSE

CSE was prepared as previously reported with some modifications [19]. Briefly, one commercial cigarette (Furong, Changde Cigarette Company, Hunan, China) was combusted with a modified syringe-driven apparatus. The smoke was bubbled through 25 ml media over 5 min by drawing 35 ml smoke every 15 s. The resulting suspension was filtered through a 0.2 μm pore-size filter to remove large particles and bacteria. This 100% CSE sample was serially diluted with PBS to obtain concentrations of 0.5%, 1%, 2.5%, and 5%, and these samples were used for the following experiments within 30 min of preparation. The optical density was consistent when comparing a series of CSE solutions prepared in this manner.

### Transient transfection of miR-34a mimic or miR-34a inhibitor

Cells were seeded into six-well plates and allowed to settle overnight until they reached 70–90% confluence. To upregulate and downregulate the expression of miR-34a, HPMECs were transfected with hsa-miR-34a mimic, specific inhibitors or miRNA-negative control (Ambion, USA) by using Lipofectamine 2000 (Invitrogen, Carlsbad, USA) following the manufacturer’s instructions. Those cells were used for experiments 48 h after transfection.

### Western blot analysis

Western blot analysis was conducted according to a previous study [20]. Briefly, the total protein in cells was extracted and measured using the BCA protein assay kit. The membrane was then washed and incubated with the respective secondary antibodies conjugated with peroxidase for 1 h. Antibody labelling was detected using enhanced chemiluminescence (ECL; Santa Cruz CA, USA). Antibodies for bax (bs-0127r), p53 (#2524 s), caspase-3 (#9662), Notch-1 (20687–1-AP, #3439), and β-actin (600008–1), as well as goat anti-mouse and goat anti-rabbit IgG horseradish peroxidase (HRP)-conjugated secondary antibodies were purchased from Proteintech Group, Inc.

### Real-time quantitative PCR (RT-qPCR)

RT-qPCR was performed using miRNA-specific primers to analyse miRNA expression. Reverse transcription PCR was performed using the Reverse Transcription System (Takara, Dalian, China). RT-qPCR was performed with iTapTM SYBR Green Supermix with ROX (Bio-Rad) using an ABI 7500 instrument (Applied Biosystems, Foster City, CA). The primers for miRNA and U6 (control) were obtained from the EzOmicsTM miRNA qPCR detection primer set (Biomics Biotechnologies, Nantong, China). Each PCR analysis was done in duplicate.

### Flow cytometry analysis

Apoptosis was analysed using Annexin V (Av) and Propidium Iodide (PI) staining (BD Biosciences, USA) [21]. Cells that were negative for Av and PI were considered to be viable. Cells that were positive for Av and negative for PI represented early apoptosis. Finally, cells that were positive for both Av and PI were classified as late apoptosis. To eliminate debris from the analysis, the discrimination level was set to 100 cells, adjusted to 1 × 105 cells per 100 μl, suspended in binding buffer and incubated with the fluorochrome-conjugated Av for 15 min. After washing, cells were re-suspended in fresh binding buffer and stained with PI. In order to set gates and establish appropriate compensation settings, cells were stained with PI alone, Av alone, and both PI and Av.

### Luciferase assay

Report constructs containing the 3′-UTR of Notch-1 were cloned into the pLUC-control vector (Promega, Madison, WI, USA). The seed sequences of miR-34a on Notch-1 were mutated using a PCR-based approach. These mutated reporter constructs were verified by sequencing. HPMECs were transiently co-transfected with the 3′-UTR reported constructs (1.5 μg/well in 6 well plates) and either hsa-miR-34a mimic or the negative control (Ambion, USA) using Lipofectamine 2000 (Invitrogen, USA). Cells were then harvested in reporter lysis buffer. Both firefly and Renilla luciferase activities were measured using the dual-luciferase assay kit (Promega, Madison, WI) according to the manufacturer’s protocol. The luciferase activity normalized against the protein concentration was expressed as a ratio of firefly luciferase to Renilla luciferase units.

### Transfection with Notch-1 plasmid

cDNA encoding a constitutively active form of Notch1 consisting of the intracellular domain (base pairs 5308–7665; amino acids 1770–2555; ICN) was subcloned into the multicloning site of the lentiviral vector pLV-EGFP/Neo to generate pLV-EGFP/Neo-EF1A-Notch-1. To generate lentiviral vectors, 10 μg The pLV-EGFP/Neo-EF1A-Notch-1 was transfected along with 7.5 μg pHelper 1.0 and 5 μg pHelper 2.0 into 293 T cells in 15 cm plates at 70% confluence for 8 h. The medium was replaced with 10 ml DMEM supplemented with 10% FBS. After culturing for 48 to 72 h, the supernatants were harvested for virus titration. The empty construct pLV-EGFP/Neo was transfected as a control. Green fluorescence was used to control the transfection efficiency. Notch-1 intracellular domain expression was confirmed by immunoblotting.

### Statistical analysis

Each experiment was replicated three times. Continuous data are presented as mean ± standard deviation (SD). Kolmogorov Smirnov analysis was performed to assess data distribution. For relative gene expression, the mean value of the vehicle control group was defined as 1 or 100%. Statistical analysis was performed using a software package (SPSS 21.0, SPSS Inc., Chicago, IL, USA). Differences between two groups were analysed using One-Way ANOVA in continuous data with Gaussian distribution. LSD test was used for post-hoc multiple comparisons. Differences/correlations between groups were compared using the Pearson’s chi-square test. A value of *p* < 0.05 was considered to be statistically significant.

## Results

### CSE-induced apoptosis in HPMECs

**Fig. 1:**
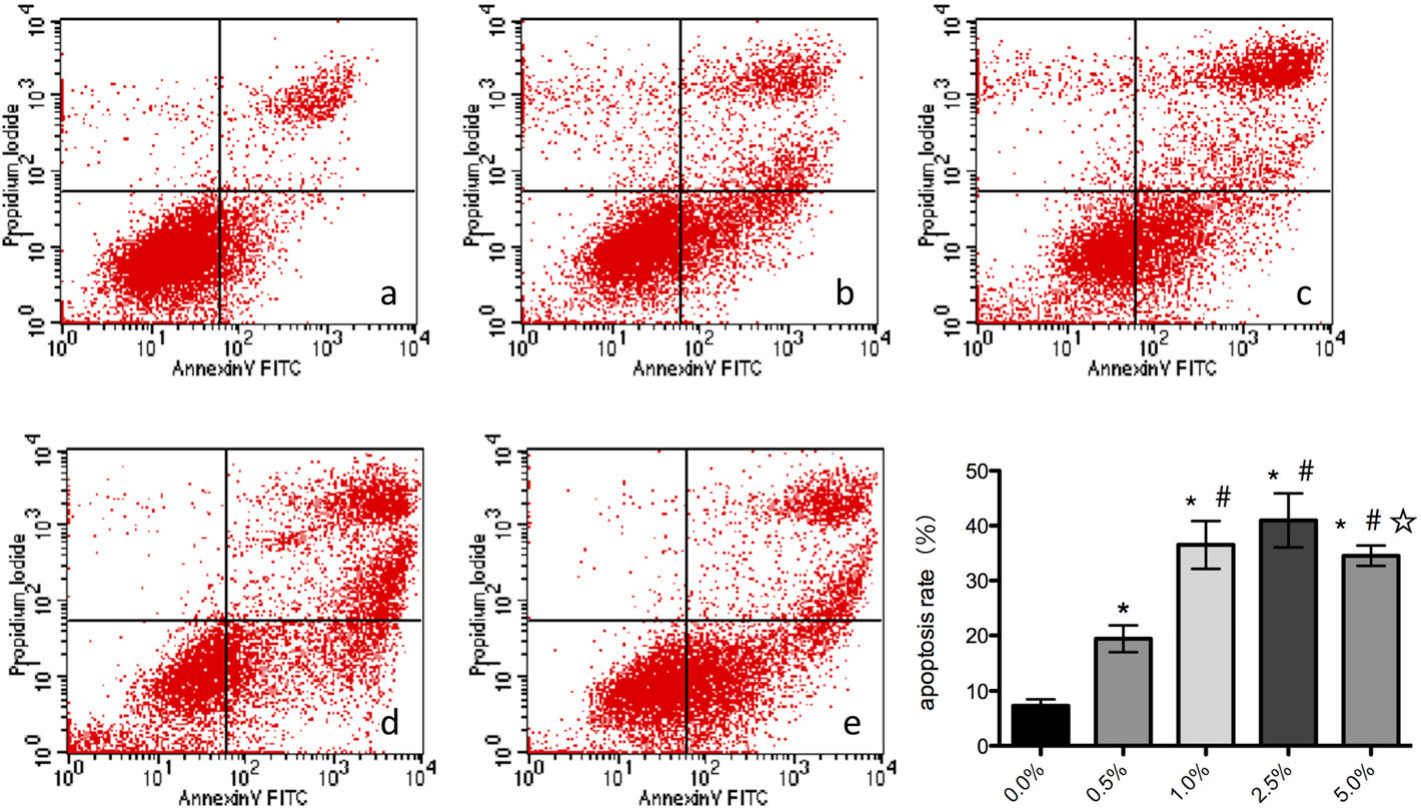
Effect of cigarette smoke extract (CSE) on apoptosis in human pulmonary microvascular endothelial cells (HPMECs). HPMECs were cultured with 0–5% CSE for 24 h. Flow cytometry analysis shows that HPMECs under exposure to varied concentration of CSE at 0.0% (**a**), 0.5% (**b**), 1.0% (**c**), 2.5% (**d**), and 5% (**e**) for 24 h respectively. Date shown represents means±SD from 3 independent experiments. ^*^P < 0.05 in compared to 0.0% of concentration. ^#^*P* < 0.05 in compared to 0.5% of concentration. ^☆^*P* < 0.05 when comparison to 2.5% of concentration

After exposing HPMECs to CSE concentrations of 0.0%, 0.5%, 1.0%, 2.5%, and 5.0% separately for 24 h, flow cytometry analysis revealed that the early apoptosis rate initially increased as CSE concentrations increased from 0.5% to 2.5%, suggesting a dose-dependent effect of CSE on apoptosis in HPMECs (Fig. 1). Specifically, the apoptosis rate of HPMECs significantly increased with exposure to 1.0% CSE (36.23 ± 4.20% by flow cytometry) in comparison to the rates at 0.0% CSE (7.33 ± 1.36%) and 0.5% CSE (19.50 ± 2.03%) (*p* < 0.05 in all). The apoptosis rate kept increasing at the 2.5% CSE (41.19 ± 5.61%), the difference remained significant. Interestingly, the early apoptosis rate deceased with exposure to 5.0% CSE (23.14 ± 1.75%), which corresponded with a significant increase in necrosis and late apoptosis rates. For subsequent experiments, we decided to expose cells to 1.0% CSE for 24 h.

### CSE upregulated the expression of miR-34a in HPMECs

**Fig. 2:**
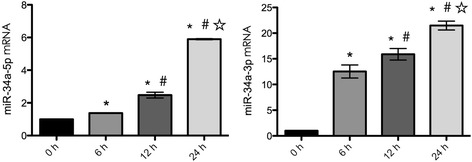
Effect of CSE on miR-34a in HPMECs. HPMECs were incubated with 1% CSE the indicated times (0–24 h) and miR-34a expression was analysed by RT-qPCR. ^*^P < 0.05 in compared to 0 h. ^#^*P* < 0.05 in compared to 6 h. ^☆^*P* < 0.05 in compared to 12 h

To gain an insight into the role of miR-34a in CSE-treated HPMECs, we first examined the expression profiles of miR-34a-5p and miR-34a-3p in HPMECs treated with CSE using RT-qPCR. The results showed that the expression of miR-34a-5p and miR-34a-3p significantly increased in HPMECs exposed to 1% CSE in an exposure time-dependent manner (Fig. 2). These results suggest a potential role for miR-34a in CSE-treated HPMECs.

### miR-34a mediates CSE induced apoptosis

**Fig. 3:**
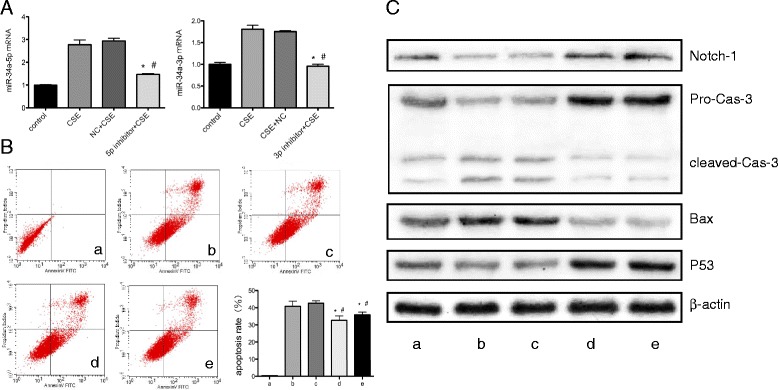
Effect of miR-34a inhibitor on CSE-treated HPMEC apoptosis rate, apoptotic proteins and downstream protein. miR-34a expression was analysed by RT-qPCR after treated with miR-34a-5p inhibitor and miR-34a-3p inhibitor (**a**). Cell apoptosis was detected by flow cytometry and statistical analysis was calculated using One-way ANOVA (**b**). Notch-1 expression and apoptotic proteins (caspase-3, bax, and P53) were detected by western blotting (**c**). HPMECs treated with 0.0% CSE were used as the control (**a**). HPMECs were treated with 1.0% CSE (b). HPMECs exposed to 1% CSE were treated with miR negative control (**c**), miR-34a-3p inhibitor (**d**), or miR-34a-5p inhibitor (**e**). CSE: cigarette smoke extract; NC: negative control mimic; 3p inhibitor: miR-34a-3p inhibitor; 5p inhibitor: miR-34a-5p inhibitor; Cas: caspase. ^*^P < 0.05 compared to CSE group. ^#^*P* < 0.05 compared to CSE plus miR negative control

To examine the effect of miR-34a on cell apoptosis, loss-of-function approaches were used. The cells were transfected with miR-34a inhibitor. We found that the inhibitor caused a decrease in miR-34a mRNA expression (Fig. 3a). CSE-treated HPMECs exhibited a significant increase in apoptosis rates, whereas miR-34a inhibitor significantly decreased CSE-induced cell apoptosis (Fig. 3b). The expression of the cleaved apoptotic proteins caspase-3 and bax significantly decreased after exposure to miR-34a inhibitor compared to control (Fig. 3c). These results imply that miR-34a is involved in regulating CSE-induced cell apoptosis in vitro. Conversely, we found that expression of p53 significantly increased in HPMECs exposed to CSE and treated with miR-34a inhibitors.

### Notch-1 is a target of miR-34a in HPMECs

To identify target genes of miR-34a, we searched for predicted target genes using the following bioinformatics algorithms: TargetScan (http://targetscan.org), miRBase (http://www.mirbase.org), and miRanda (http://www.microrna.org). The three algorithms identified multiple target genes, including Notch-1, that are associated with endothelial cell proliferation and apoptosis.

**Fig. 4:**
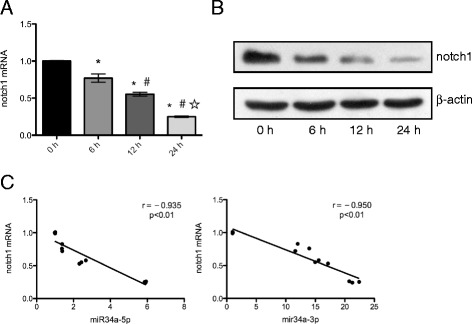
Effect of CSE on Notch-1 in HPMECs and correlation between miR-34a and Notch-1. Notch-1 expression in HPMECs treated with CSE (**a**, **b**) is negatively correlated with miR-34a expression (**c**). Statistically significant inverse correlation between miR-34a and Notch-1 in HPMECs treated with 1% CSE at the indicated times (0–24 h) (Spearman’s correlation analysis, miR-34a-5p: *r* = − 0.935; miR-34a-3p: *p* < 0.01, *r* = − 0.950; *p* < 0.01). ^*^P < 0.05 compared to 0 h exposure. ^#^*P* < 0.05 compared to 6 h exposure. ^☆^*P* < 0.05 compared to 12 h exposure

We used RT-PCR, western blot analysis, and the luciferase reporter assay to determine whether Notch-1 was regulated by miR-34a. As expected, the expression level of Notch-1 was substantially decreased in HPMECs exposed to CSE (Fig. 4a and Fig. 4b). Spearman’s correlation test revealed a negative correlation between miR-34a relative expression and Notch-1 expression (*r* = − 0.935 and *r* = − 0.950, respectively) (Fig. 4c). The miR-34a inhibitor was able to restore the expression of Notch-1 in CSE-treated HPMECs (Fig. 3c).

**Fig. 5:**
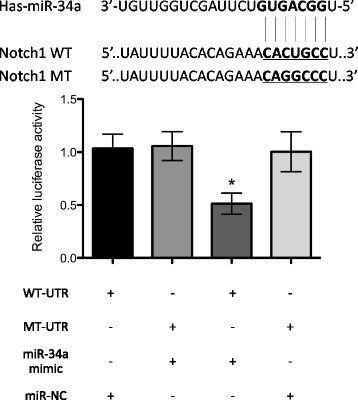
Luciferase report gene demonstrates that miR-34a directly targets the 3’ UTR of Notch-1. Cells were transfected with pLUC reporter plasmids containing either the wild-type (WT) or mutant type (MT) of the 3′UTR of Notch-1 in the presence of miR-34a mimic or negative control, and cultured for 48 h before being harvested for analysis. miR negative control was used as the control. miR-34a mimic repressed the activity of the wild-type Notch-1 3′-UTR, but not that of the mutant constructs. *P < 0.05 compared to miR negative control group

Finally, we constructed a luciferase reporter assay to verify that Notch-1 is the direct target of miR-34a. We transfected HPMECs with miR-34a mimic and scrambled miRNA (negative control). The targeting of miR-34a to the 3′-UTR of Notch-1 mRNA was examined using luciferase constructs that were cloned into the pLUC-control vector. With wild-type Notch-1 3e–UTR, luciferase activity decreased following ectopic miR-34a expression (*p* < 0.05); however, this effect was not observed in the mutant constructs (Fig. 5). Collectively, these results suggest that Notch-1 is negatively regulated by miR-34a in HPMECs.

### Restoration of Notch-1 can rescue the effects of miR-34a in CSE-treated HPMECs

**Fig. 6:**
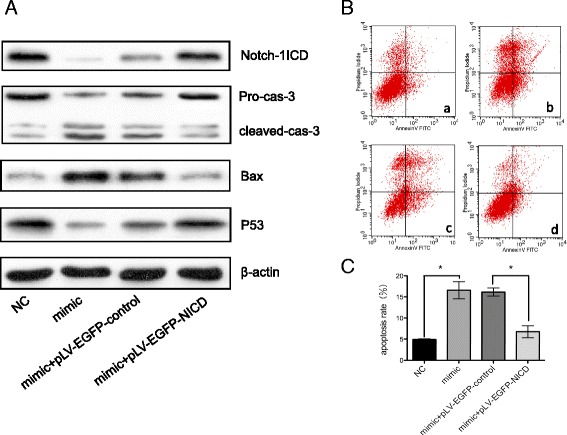
Notch-1 attenuates miR-34a induced apoptosis of HPMECs. Notch-1 intracellular domain and apoptotic proteins (caspase-3, bax, and p53) were detected by western blotting (**a**). Flow cytometry analysis shows the rate of HPMEC apoptosis under different conditions (**b**). Statistical analysis was calculated (**c**). HPMECs treated with miR negative control (**a**), miR-34a mimic (**b**), blank vector (**c**), or NICD (d). NC: negative control; mimic: miR-34a mimic; Cas: caspase; ^*^P < 0.05 compared to control

To deeply investigate the role of Notch-1 and miR-34a in apoptosis of HPMECs, we upregulated the expression of miR-34a by transfecting with miR-34a mimics in HPMECs. When miR-34a mimics were transfected into HPMECs, the level of miR-34a increased up to 3.7-fold compared with control group. The expression levels of Notch-1 intracellular domain were determined 24 h after pLV-EGFP-NICD or pLV-EGFP-control vector was transfected into cells. As shown in Fig. 6a, pLV-EGFP-NICD rescued the increased apoptotic protein expression in HPMECs treated with miR-34a mimic, and led to a corresponding induction of p53 levels. In addition, transfection with NICD expression yielded a protective effect from apoptosis (Fig. 6b). Statistical significance between apoptotic rates was calculated compared with control (Fig. 6c). These results suggest that restoration of Notch-1 can rescue the effect of miR-34a-induced apoptosis in HPMECs.

## Discussion

Studies in human subjects and experimental studies in animal models of COPD provide a great amount of insight into the association between cigarette smoke, cellular apoptosis, and the development of emphysema. Previously, we demonstrated that apoptosis is involved in alveolar epithelial cells and endothelial cells in the parenchyma of COPD-afflicted tissues in both animal models and human clinical studies [8–10, 22]. As the most important risk factor of COPD, cigarette smoke can initiate apoptosis in fibroblasts, macrophages, alveolar epithelial cell lines, and vascular endothelial cells [23–25]. Consistent with this concept, the present study demonstrates that CSE induces apoptosis in HPMECs in a dose-dependent manner. Several mechanisms have been proposed as being responsible for CSE-mediated cell apoptosis, including the accumulation of misfolded proteins in the endoplasmic reticulum and alterations in signalling pathways. Although some studies have proposed that CSE can directly induce cell apoptosis or necrosis, the associated process may depend on the concentration or the exposure time of CSE. Low concentrations or short exposure time may induce cell apoptosis, whereas high concentrations or long exposure time may instead induce necrosis [3]. We also found that early apoptosis rates decreased at high concentrations of CSE, whereas necrosis and late apoptosis rate increased in this condition. To better understand the mechanisms regulating HPMECs apoptosis, we opted to apply standard conditions (1% CSE for 24 h) leading to high early apoptosis rate and moderate necrosis for subsequent studies. Our results strongly suggest that HPMEC apoptosis induced by cigarette smoke may contribute to the pathogenesis of COPD.

miRNAs have recently been implicated in COPD pathogenesis [26]. miRNAs are small noncoding RNAs of 19–25 nucleotides that can cause post-transcriptional gene repression either by increasing mRNA degradation or by inhibiting protein translation of specific mRNA targets. It is well established that miRNAs impair all known cellular and developmental processes. Dysregulation of miRNAs have been widely shown to be associated with the development of smoking-related pulmonary diseases. Several studies have reported that miRNAs are differentially expressed in different tissue types obtained from COPD patients compared to control groups [27–29]. Multiple studies using a variety of models have demonstrated that direct exposure to cigarette smoke or exposure to cigarette smoke extract affects miRNA expression in the lungs [30–32]. Persistent smoking-related changes in miRNAs may play a role in the subsequent development of disease, even after smoking cessation [33]. The exact mechanism by which this dysregulation causes COPD remains unknown. In the present study, we found that miR-34a expression is significantly increased and is involved in CSE-induced apoptosis of HPMECs. MiR-34a belongs to one of several evolutionarily conserved families of miRNAs (the miR-34 family), and was originally identified as a *TP53*-targeting miRNA [34]. Numerous studies have demonstrated that miR-34a is critically involved in regulating cell apoptosis and cell cycle [35, 36]. Previous studies have reported an increased expression of miR-34a in the lungs of COPD patients compared with control groups and a strong correlation between miR-34a and FEV1% [14]. Our in vitro studies have shown that miR-34a is involved in apoptosis of HPMECs. Inhibiting the expression of miR-34a remarkably attenuated the expression of Bax and cleaved caspase-3 which are indicator for cell apoptosis in the presence of CSE. These results suggest that miR-34a may contribute to apoptosis of endothelial cells. However, whether the observed effect in HPMEC is specific for cigarette induced apoptosis requires further investigation. MiRNAs modulate biological functions by targeting multiple mRNAs. Identifying functionally important mRNA targets of miR-34a is essential to unravelling its biological function and is helpful for further investigation.

Computational miRNA target prediction suggested that Notch-1 is a target of miR-34a. A direct interaction between Notch-1 and miR-34a has already been reported [37]. In the present study, an increase in miR-34a expression is associated with a decrease in Notch-1 expression in HPMECs treated with CSE. A luciferase reporter assay further demonstrated that miR-34a binds to the putative 3′-UTR-binding sites of Notch-1, which is a necessary event for miRNA to elicit its biological function. Notch-1 receptor protein serves as one of the four receptors (Notch-1, 2, 3, and 4) in Notch signalling, originally discovered in Drosophila. The notch family of transmembrane receptors are important regulators of cell fate determination events in different cell types. Upon activation by ligands usually located on the surface of neighbouring cells, Notch undergoes intra-membrane proteolysis, resulting in the release of an intracellular region that stimulates the downstream signalling cascade [38]. Early studies revealed that the Notch pathway regulates the development of airway epithelium, mesenchymal stroma, and pulmonary vasculature, and down-regulation of the Notch pathway in human airway epithelium has been associated with smoking and COPD [39–41]. Our previous study has demonstrated that Notch-1 was downregulated in lung tissue of COPD compared to those of non-COPD smokers as well as non-smokers [42]. Furthermore, Notch-1 has been shown to protect against anoikis (apoptosis induced by matrix withdrawal) or p53-mediated apoptosis in immortalized epithelial cells [43, 44], T-cell receptor-induced apoptosis in mature cells [45], and dexamethasone-mediated apoptosis in thymocytes [46]. The miR-34a/Notch-1 axis, which affects cell proliferation and apoptosis, has been widely studied for its role in malignant diseases [47, 48]. However, the association with COPD remains unclear and requires further investigation. In the present study, we observed the inverse correlation between miR-34a and Notch-1 in HPMECs treated with CSE. Moreover, transfection of the intracellular domain of Notch-1 plasmid nullified the effect of miR-34a on cellular apoptosis, indicating that Notch-1 is involved in miR-34a induced apoptosis in HPMECs. Among other Notch receptors, Notch-2 is approved to be another target of miR-34a using bioinformatics algorithms. However, previous study revealed that no differences were seen in expression of Notch-2 among normal nonsmokers, normal smokers and smokers with COPD [42].

The expression of the apoptotic proteins caspase-3 and bax significantly decreased after treatment with an inhibitor specific for miR-34a compared to the CSE control group; however, this effect was not observed for p53 expression. Previous studies showed that p53 activates miR-34a expression, and that miR-34a increased the activity of p53, suggesting the existence of a p53-miR34 positive feedback loop [49]. Our results provide further evidence for this feedback loop.

Considerable evidence supports a multidirectional and complex interaction between Notch and p53. Notch signalling can either suppress or increase p53 activity in a context-dependent manner. Notch-1 inhibits p53 activity in cervical cancer cells [44]. Previous research indicated that in cell line of hepatoma, colon, prostate and breast origin, Notch-1 inhibits p53 via the mTOR pathway [50]; however, Notch can activate p53 in hepatocellular cells as a propoptotic factor [51] and during early embryonic development [52]. On the other hand, p53 can also regulate Notch, and positive and negative feedback loops have been reported between p53 and Notch [53]. p53 induces Notch expression in keratinocytes, stimulating differentiation and preventing tumour formation [54], whereas, p53 suppresses Notch activation in murine thymoma cell line [55]. The data we present here supports the role of Notch-1 as a positive regulator of p53 in HPMECs.

## Conclusion

In summary, our results suggest that miR-34a is involved in CSE-induced apoptosis of HPMECs. Furthermore, this effect may be associated with the regulation of its target, Notch-1, in endothelial cell lines. The present study reveals that miR-34a may be a key regulator of cellular apoptosis and a potential therapeutic target in future.

## Abbreviations

3′-UTR: 3′-untranslated region
COPD: Chronic obstructive pulmonary disease
CSE: Cigarette smoke extract
HPMECs: Human pulmonary microvascular endothelial cells
miR-34a: microRNA-34a
